# Single mutations to tyrosine or glutamate improve the crystallizability and crystal diffraction properties of a flexible two-domain protein

**DOI:** 10.1107/S2053230X25010416

**Published:** 2026-01-01

**Authors:** Christina Geerds, Hartmut H. Niemann

**Affiliations:** a Department of Chemistry Bielefeld University Universitätsstrasse 25 33615Bielefeld Germany; Centro Nacional de Biotecnología – CSIC, Spain

**Keywords:** multidomain proteins, point mutations, protein crystallization, surface-entropy reduction, surface mutation, crystal contacts

## Abstract

This case report describes single surface substitutions that improve the crystallizability and diffraction properties of a flexible two-domain protein. InlB_392_ comprises the internalin domain and the B repeat of the *Listeria monocytogenes* invasion protein InlB. The InlB_392_ wild type yielded very few poorly reproducible hits in crystallization screens and the crystals had a diffraction limit of worse than 3.0 Å. It seems reasonable to assume that this crystallization bottleneck is caused by interdomain flexibility, given that crystals of the isolated internalin domain or B repeat diffract to high resolution. A previously identified variant, T332E, showed improved crystallization and diffraction. Here, two additional InlB_392_ variants are described with single threonine-to-tyrosine or valine-to-glutamate substitutions that produced crystals directly in initial screens and, without optimization, diffracted to 1.6 and 1.45 Å resolution, respectively. The mutated residues do not participate in intramolecular interdomain interactions but mediate crystal contacts, indicating that specific surface properties, rather than interdomain flexibility *per se*, impede the crystallization of wild-type InlB_392_. Notably, the beneficial glutamate substitutions contrast with the generally recognized underrepresentation of glutamate in crystal contacts and the high entropic cost of fixing an otherwise flexible side chain with many rotatable bonds in a crystal contact. The reported results suggest that surface mutations can help crystallization even if they increase the entropy of the respective residue. More broadly, the observations are consistent with the hypothesis that negative evolutionary design limits fortuitous lattice formation of proteins and the resulting expectation that random mutations of surface residues are likely to improve crystallizability.

## Abstract 2

In its wild-type form, the flexible two-domain protein InlB_392_ yielded poorly reproducible crystals that diffracted to low resolution. Single surface substitutions to tyrosine (T336Y) or glutamate (V333E) yielded well diffracting crystals, suggesting that surface properties, rather than interdomain flexibility, might be the primary impediment to crystallization of the wild type.

## 1. Introduction

Many factors, including the composition of the reservoir solution and physical parameters such as the temperature, influence the crystallization of a given protein (McPherson & Gavira, 2014 ▸), but the protein sample itself is often the main determinant for success (Longenecker *et al.*, 2001 ▸; Dale *et al.*, 2003 ▸). Both the covalent and conformational heterogeneity of the protein should be low (Deller *et al.*, 2016 ▸). Conformational heterogeneity includes interdomain flexibility caused by mobile linkers in multidomain proteins and surface heterogeneity arising from flexible termini or internal loops, from glycosylation and from the side chains of surface-exposed high-entropy residues such as lysine or glutamate. Several strategies exist to mitigate these issues (Ruggiero *et al.*, 2012 ▸; Deller *et al.*, 2016 ▸). Flexible termini and loops can be removed using recombinant methods (Derewenda, 2004*b* ▸; Gäfe & Niemann, 2023 ▸), by limited proteolysis (Geerds *et al.*, 2014 ▸) or by *in situ* proteolysis (Dong *et al.*, 2007 ▸; Wernimont & Edwards, 2009 ▸; Tong *et al.*, 2014 ▸; Horstmeier *et al.*, 2025 ▸). Crystallization chaperones that simultaneously bind to at least two domains can reduce interdomain flexibility (Koide, 2009 ▸; Bukowska & Grütter, 2013 ▸). Nanobodies have been particularly successful as they can insert into concave regions to stabilize the hinges between domains (Desmyter *et al.*, 2015 ▸). Covalent microheterogeneity and conformational surface heterogeneity due to glycosylation can be reduced by mutation (Derewenda, 2004*b* ▸) or by using glycosylation-deficient cell lines, often in combination with enzymatic deglycosylation (Chang *et al.*, 2007 ▸; Niemann *et al.*, 2007 ▸). Surface-entropy reduction (SER), which replaces high-entropy side chains on the protein surface with smaller, less flexible amino acids, has become a widely employed rescue procedure for target proteins that yield no or poor-quality crystals (Derewenda, 2004*a* ▸; Goldschmidt *et al.*, 2014 ▸; Barden *et al.*, 2013 ▸; Koopmeiners *et al.*, 2024 ▸).

InlB_392_, a C-terminally truncated version of InlB, is one example of a protein that has so far been difficult to crystallize. InlB is a five-domain surface-located invasion protein that facilitates the uptake of pathogenic *Listeria monocytogenes* into host cells by activating the human receptor tyrosine kinase MET (Bierne & Cossart, 2002 ▸; Niemann, 2013 ▸; Ireton *et al.*, 2021 ▸). The introduction of a recent paper summarizes the structure–function relationship of its domains (Geerds *et al.*, 2022 ▸).

InlB_392_ comprises the first two domains of InlB: the internalin domain (residues 36–320) and the B repeat (residue 321–392; the residue numbering follows that of full-length InlB). Each domain yielded well diffracting crystals with resolution limits of 1.6 and 1.3 Å (Schubert *et al.*, 2001 ▸; Ferraris *et al.*, 2010 ▸; Ebbes *et al.*, 2011 ▸). Our first attempt to obtain a structure of InlB_392_ resulted in crystals that diffracted to 3.2 Å resolution, in which the internalin domain mediated all packing contacts and there was no electron density for the B repeat (Ebbes *et al.*, 2011 ▸). This is most likely due to interdomain flexibility, because the C-terminus of the internalin domain points into large solvent channels that can accommodate the B repeat in various orientations (Ebbes *et al.*, 2011 ▸). In crystals of full-length InlB, the electron density was not sufficient to model the B repeat, although the three C-terminal GW domains could be built (Marino *et al.*, 2002 ▸). The susceptibility of the B repeat to proteolysis supported a flexible linkage to the internalin domain (Marino *et al.*, 2002 ▸). Only recently, we obtained structures of InlB_392_ in which the B repeat was resolved (Geerds *et al.*, 2022 ▸). These structures showed almost no stabilizing interactions between the internalin domain and the B repeat, and they confirmed the high interdomain flexibility. These observations suggested that interdomain flexibility might be the main impediment to crystallization.

One finding speaks against this hypothesis. In our second attempt to crystallize InlB_392_, the wild type gave crystals in a single condition. These crystals were not reproducible and diffracted to only 3.3 Å resolution (Geerds *et al.*, 2022 ▸). In contrast, a single substitution in the B repeat, T332E, facilitated crystallization under multiple conditions despite increasing the local surface entropy. So far, we have determined structures of InlB_392__T332E at 2.05 and 1.8 Å resolution in two crystal forms (Geerds *et al.*, 2022 ▸). This suggested that specific surface properties, rather than interdomain flexibility, might be the primary impediment to the crystallization of wild-type InlB_392_. Here, we report the crystallization and the high-resolution structures of two additional InlB_392_ variants with single substitutions in the B repeat which increase the surface entropy. These results support the hypothesis that conformational heterogeneity may be overcome by the introduction of specific crystal contacts to allow the growth of well diffracting crystals of wild-type InlB_392_.

## 2. Materials and methods

### 2.1. Macromolecule production

Both variants (Table 1 ▸) were produced essentially as described by Bleymüller *et al.* (2016 ▸). Briefly, the proteins were expressed as glutathione *S*-transferase (GST) fusions in *Escherichia coli* at 20°C overnight. Cleared cell lysate in phosphate-buffered saline was applied onto a glutathione affinity matrix. The target protein was liberated from the GST-tag by cleavage with human rhinovirus 3C protease and was further purified by anion-exchange chromatography (Source Q; GE Healthcare). For crystallization, the buffer was exchanged to 10 m*M* Tris pH 8.0, 20 m*M* NaCl.

### 2.2. Crystallization

Initial crystallization trials were set up with the 96-condition MORPHEUS screen (Gorrec, 2009 ▸) and the 192-condition PEG smear screen (Chaikuad *et al.*, 2015 ▸) at 4 and 20°C using drop ratios of 100 nl protein solution + 100 nl reservoir solution and 200 nl protein solution + 100 nl reservoir solution. Crystallization conditions for the crystals for which we report structures are given in Table 2 ▸. The T336Y variant crystallized as bipyramids. Crystals appeared within 3–14 days and were flash-cooled in liquid nitrogen without additional cryoprotection. The crystal used for structure determination was harvested from a drop set up with the original solution from the commercial screen using 1 µl + 1 µl drops and measured approximately 280 × 60 × 60 µm. Crystals of the V333E variant were detected about five weeks after setup. They were cryoprotected in 0.1 *M* HEPES pH 7.5, 23% PEG smear medium molecular-weight mixture (MMW; Chaikuad *et al.*, 2015 ▸) containing 15% glycerol and flash-cooled in liquid nitrogen. The crystal used for structure determination measured approximately 90 × 20 × 20 µm.

### 2.3. Data collection and processing

Data for the T336Y variant were collected on beamline BL14.2 at the BESSY II electron-storage ring operated by the Helmholtz-Zentrum Berlin (Mueller *et al.*, 2015 ▸). Data for the V333E variant were collected on beamline P13 operated by EMBL Hamburg at the PETRA III storage ring (Cianci *et al.*, 2017 ▸). Both data sets were indexed and integrated with *XDS* (Kabsch, 2010 ▸) and scaled with *XSCALE* using zero-dose extrapolation (Diederichs *et al.*, 2003 ▸). Data-collection and processing statistics are summarized in Table 3 ▸.

### 2.4. Structure solution and refinement

Both structures were solved by molecular replacement using *Phaser* (version 2.8.3; McCoy *et al.*, 2007 ▸) run through *ccp*4*i* (Potterton *et al.*, 2003 ▸). The internalin domain (PDB entry 1h6t) and the B repeat (PDB entry 2y5p; chain *A*) were placed sequentially. For the T336Y variant, running *Phaser* with default options (tNCS enabled) resulted in a long CPU time and 11 potential solutions. Disabling tNCS yielded a single solution. The domains were rearranged in *Coot* (Emsley *et al.*, 2010 ▸; Casañal *et al.*, 2020 ▸) to obtain three complete InlB_392_ molecules and this assembly was matched to the InlB_392_ wild-type structure (PDB entry 7pv9) with *CSYMMATCH* from the *CCP*4 suite (Agirre *et al.*, 2023 ▸). Both structures were rebuilt in *Coot* and refined initially with *REFMAC*5 (Kovalevskiy *et al.*, 2018 ▸) and during the later stages with *phenix.refine* (Liebschner *et al.*, 2019 ▸) using TLS refinement and riding hydrogens. Refinement statistics are given in Table 4 ▸.

## 3. Results

### 3.1. Crystallization propensity of InlB_392_ variants

**Figure 1:**
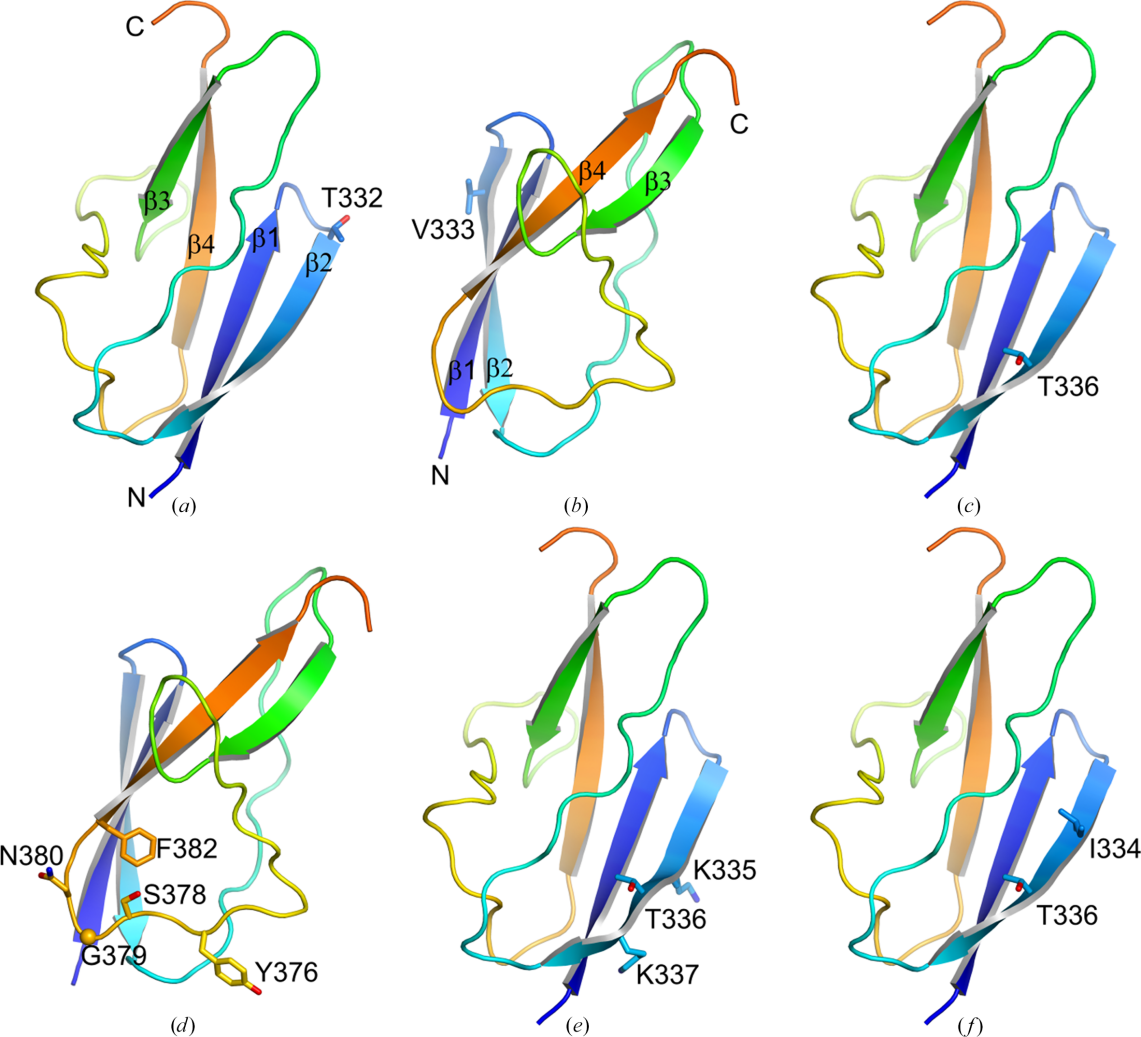
Locations of the mutations in the six InlB_392_ variants. Only the B repeat (residues 321–392) is shown as a cartoon. N- and C-termini and secondary-structure elements are labeled in (*a*) and (*b*). Mutated residues are shown as sticks (Gly as a C^α^ sphere). (*a*), (*c*), (*e*) and (*f*) share the same orientation; (*b*) and (*d*) show a different view. (*a*) T332E. (*b*) V333E. (*c*) T336Y. (*d*) Variant A (Y376K, S378P, G379T, N380K, F382I). (*e*) Variant C (K335S, T336K, K337E). (*f*) Variant D (I334K, T336L).

We had obtained initial crystals of InlB_392_ from the commercial screens JCSG Core I–IV, MBClass, PACT and PEGs (672 conditions in total), but there was no electron density for the B repeat in the resulting structure (Ebbes *et al.*, 2011 ▸). Therefore, we tested new crystallization screens, namely MORPHEUS and PEG smear (Gorrec, 2009 ▸; Chaikuad *et al.*, 2015 ▸), with wild-type InlB_392_ and six InlB_392_ variants with single or multiple substitutions in the B repeat. These mutations had originally been chosen to map a presumed protein–protein binding site but not to rationally improve the crystallization propensity (Bleymüller *et al.*, 2016 ▸). All mutations except variant A are located in strand β2 of the B repeat (Fig. 1 ▸).

We screened these proteins under similar but not identical conditions (Table 5 ▸). Although this study was neither systematic nor extensive enough to draw general conclusions, the outcomes provide a qualitative comparison of crystallization propensity. Wild-type InlB_392_ crystallized under a single condition at 4 and 20°C; the crystals were not reproducible and yielded a 3.3 Å resolution structure (Geerds *et al.*, 2022 ▸). Two variants with multiple substitutions (variant A, Y376K, S378P, G379T, N380K, F382I; variant C, K335S, T336K, K337E) yielded no crystals. Variant D (I334K, T336L) crystallized under many conditions, mainly in the PEG smear screen but also in MORPHEUS. These crystals were predominantly needles and most diffracted to worse than 4 Å resolution, with the best at 3.3 Å (the mean resolution for nine data sets was 5.04 Å). We have not pursued this structure so far. The most successful variants contained single substitutions. The T332E variant crystallized under many conditions across screens and temperatures. So far, we have published structures of two crystal forms at 2.05 and 1.8 Å resolution (Geerds *et al.*, 2022 ▸). We had also collected data sets from InlB_392_ T332E variant crystals grown under different conditions. Some of these were isomorphous to crystal form I (PDB entry 7pv8). For the other crystals, we have not yet been able to solve or satisfactorily refine the structures, presumably due to symmetry problems such as pseudotranslation and/or twinning (Geerds *et al.*, 2022 ▸). The present work focuses on the two additional single substitutions V333E and T336Y.

### 3.2. Crystallization and structure determination

Due to the limited amount, the V333E variant was only tested in the 192-condition PEG smear screen containing low-molecular-weight (LMW), medium-molecular-weight (MMW), high-molecular-weight (HMW) and broad-molecular-weight (BMW) PEG mixtures at 4 and 20°C, and crystallized in one condition at 4°C. The T336Y variant was tested in the MORPHEUS and PEG smear (LMW and BMW at 4 and 20°C, HMW and MMW at 20°C) screens and crystallized in three MORPHEUS conditions and two PEG smear conditions.

For the V333E variant, we collected five data sets from PEG smear MMW condition E1 (Table 2 ▸) with resolution limits between 2.2 and 1.45 Å. These crystals shared the space group of crystal form II of the T332E variant (PDB entry 7nms), with about 2 Å deviation in the lengths of all three unit-cell axes, and contained one molecule per asymmetric unit. Molecular replacement was straightforward.

For the T336Y variant, we collected data sets from one crystal from MORPHEUS H3 (2.1 Å resolution), five crystals from MORPHEUS F3 (1.6–1.85 Å resolution) and nine crystals from PEG smear MMW E1 (1.95–3.2 Å resolution). All crystals had the same space group as wild-type InlB_392_ (PDB entry 7pv9), with variations of up to 4 Å in the *b* and *c* axes, and contained three molecules per asymmetric unit. A strong off-origin peak (32% of the origin peak) was present in the native Patterson map at (*u*, *v*, *w*) = (0, 0.321, 0), consistent with tNCS. For the best performance, the tNCS option of *Phaser* was turned off. Data-collection and refinement statistics are reported in Tables 3 ▸ and 4 ▸.

### 3.3. Overall structure and interdomain flexibility

**Figure 2:**
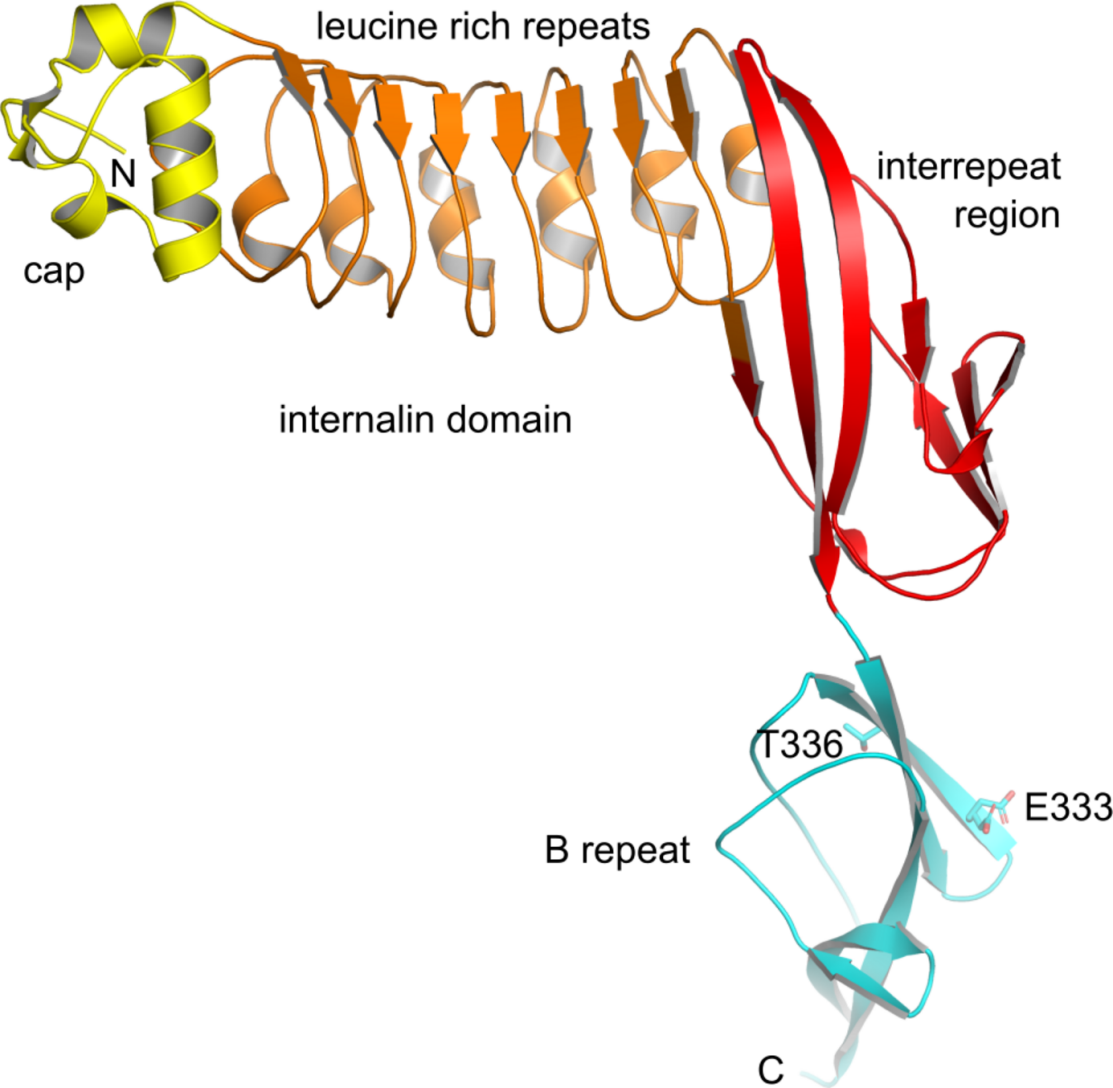
Structure of the InlB_392_ V333E variant. Cartoon representation showing the internalin domain subdivided into the capping structure (yellow), leucine-rich repeats (orange) and the interrepeat region (red); the B repeat is cyan. Side chains at positions 333 and 336 are shown as sticks; in the V333E variant residue 336 is threonine (wild type) and Glu333 is modeled in two conformations. N- and C-termini are labeled.

Fig. 2 ▸ provides an overview of the the highest resolution structure (V333E). The overall protein structure closely matches previous InlB_392_ structures (wild type and T332E; Geerds *et al.*, 2022 ▸).

**Figure 3:**
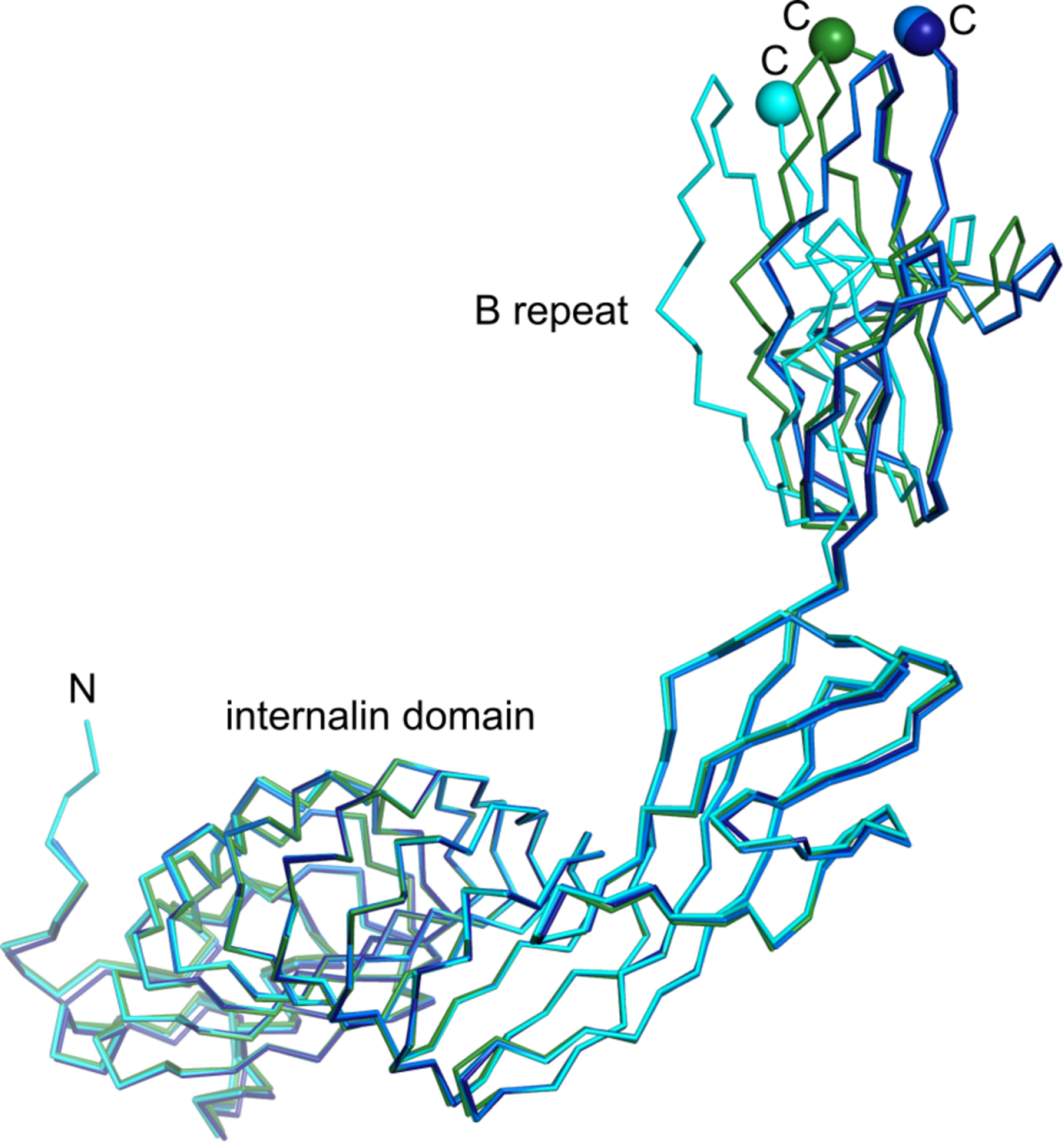
Interdomain flexibility between the internalin domain and the B repeat. The four crystallographically independent chains reported here are superposed on the internalin domain to highlight relative B-repeat motions. The InlB_392_ V333E variant is shown in green and InlB_392_ T336Y chains *A*, *B* and *C* are shown in dark blue, blue and cyan, respectively. N and C-termini are labeled. C^α^ atoms of the C-terminal residues are shown as spheres.

The four crystallographically independent chains described here (three in the T336Y structure and one in the V333E structure) again show substantial interdomain flexibility between the internalin domain and the B repeat (Fig. 3 ▸). The relative domain orientation of T336Y chains *A*–*C* resembles that of wild-type chains *A*–*C* (Supplementary Figs. S1*a*–S1*c*). The orientation of the V333E variant is closest to crystal form II (PDB entry 7nms) of the T332E variant (Supplementary Fig. S1*d*).

### 3.4. Crystal packing

**Figure 4:**
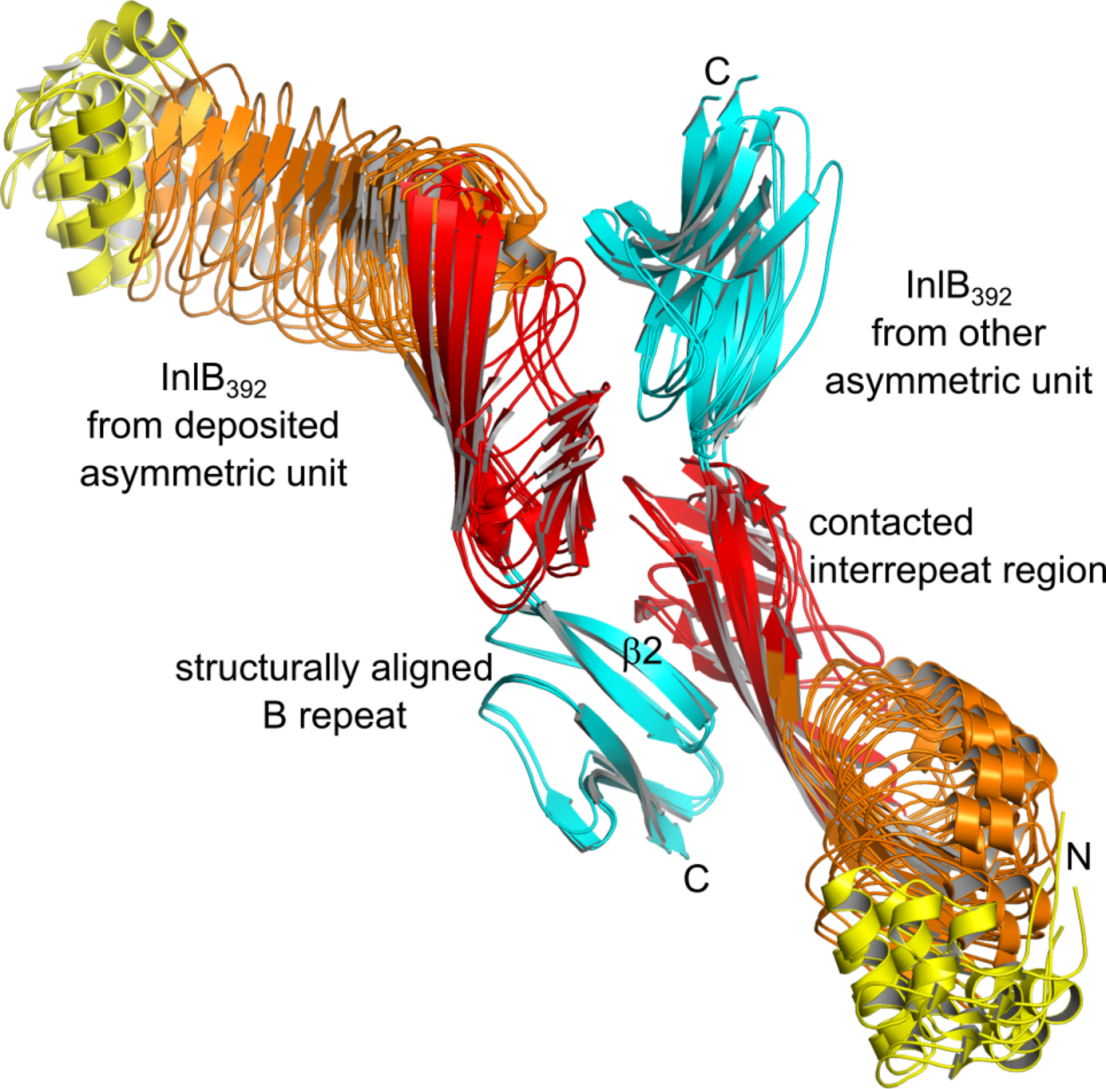
A conserved crystal contact present in all InlB_392_ structures. The coloring follows Fig. 2 ▸ (internalin domain, yellow/orange/red; B repeat, cyan). The four independent chains reported here are superposed on the B repeat (left molecule). All four form a crystal contact between strand β2 of the B repeat and the interrepeat region (red) of a neighboring internalin domain (right molecule). The dimeric arrangement shown is not *C*_2_-symmetric. The B repeat of the right molecule forms the same contact with another neighbor.

We had previously found all available InlB_392_ structures with a resolved B repeat (PDB entries 7nms, 7pv8 and 7pv9) to share a recurring crystal contact between strand β2 of the B repeat and the interrepeat region of a neighboring internalin domain (Geerds *et al.*, 2022 ▸). With the same space group and similar unit-cell constants, the packing of the T336Y variant is virtually identical to that of the wild type (PDB entry 7pv9), and the packing of the V333E variant is virtually identical to that of crystal form II of the T332E variant (PDB entry 7nms). Accordingly, all three chains of the T336Y variant and the V333E variant chain also form the recurring crystal contact (Fig. 4 ▸).

**Figure 5:**
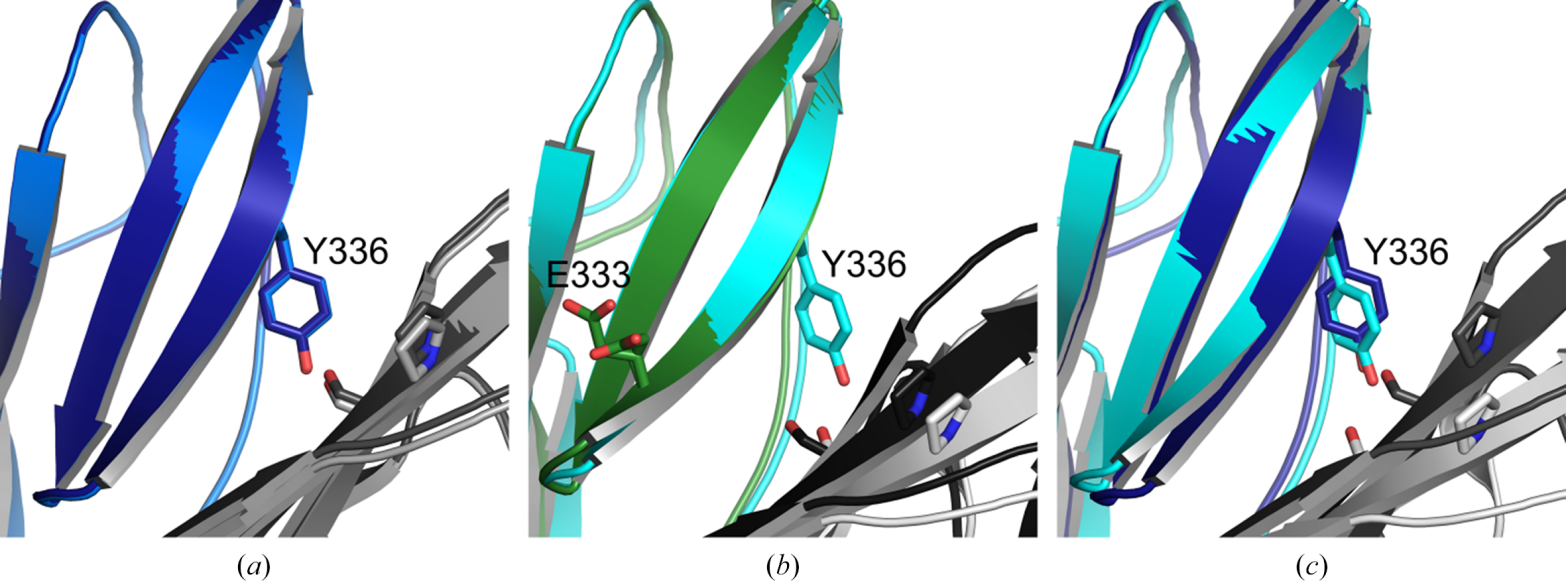
Two geometries of the recurring crystal contact. Contact between the B repeat of one InlB_392_ molecule (left; colored) and the interrepeat region of a neighboring molecule (right; grayscale). Side chains of the mutated residues (Glu333 and Tyr336) are shown as sticks. In the neighboring interrepeat region, Ser295 and Pro318 are shown as sticks to highlight aligned or shifted β-strand positions. (*a*) Overlay of T336Y chain *A* (dark blue) on chain *B* (blue) and their contacting neighbors (dark gray and gray). (*b*) Overlay of the V333E variant (green) on T336Y chain *C* (cyan) and their contacting neighbors (black and light gray). (*c*) Overlay of T336Y chains *A* (dark blue) on *C* (cyan) and their contacting neighbors (dark gray and light gray).

In both the T336Y and V333E variants, the substituted residue lies within this interface. The contacts made by T336Y chains *A* and *B* are very similar to each other (Fig. 5 ▸*a*), as are those made by V333E and T336Y chain *C* (Fig. 5 ▸*b*). Comparing these two groups reveals a shift of the interrepeat region of the symmetry mates when the B repeats are superposed (Fig. 5 ▸*c*).

**Figure 6:**
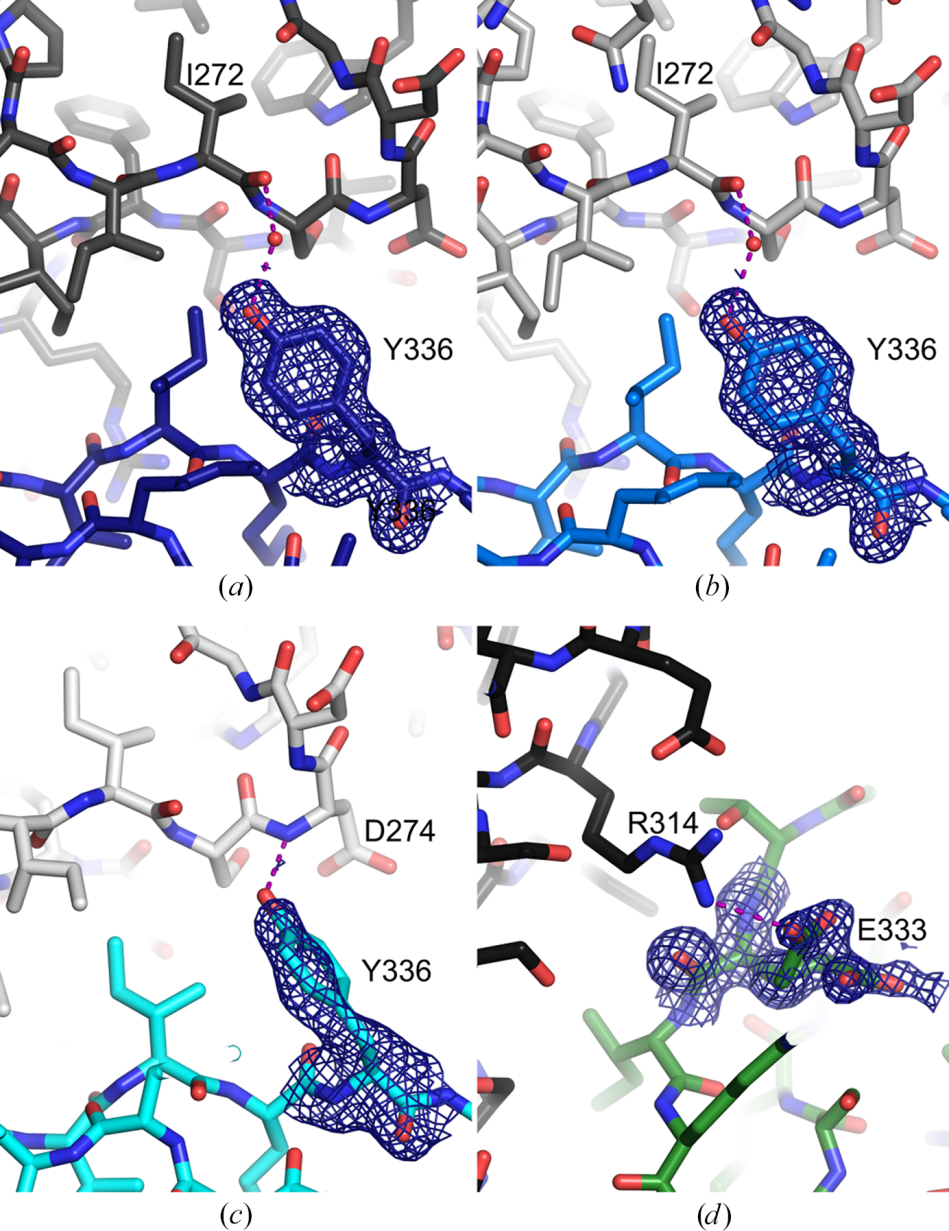
Mutated residues within the crystal contact and their electron density. The B repeats of T336Y chains *A*, *B* and *C* are shown in dark blue, blue and cyan, respectively; the V333E variant is in green. The interrepeat region of the neighboring molecule is shown in grayscale. For the mutated residues, 2*mF*_o_ − *DF*_c_ electron-density maps are contoured at 1.0σ. Hydrogen bonds and the salt bridge involving the mutated side chains are shown as purple dashed lines. (*a*) T336Y chain *A*. (*b*) T336Y chain *B*. (*c*) T336Y chain *C*. (*d*) V333E; Glu333 is modeled in two conformations.

Tyr336 in T336Y chains *A* and *B* is well ordered and forms a water-mediated hydrogen bond to the Ile272 carbonyl of a neighboring molecule (Figs. 6 ▸*a* and 6 ▸*b*). In chain *C*, Tyr336 is less well ordered, but may hydrogen-bond to the backbone NH of Asp274 of a symmetry mate (Fig. 6 ▸*c*). Glu333 in the V333E variant adopts two conformations, which we modeled with about 50% occupancy each. One points into solvent, while the other forms an intermolecular salt bridge with Arg314 of a neighboring molecule (Fig. 6 ▸*d*). Glu332 in the T332E variant also forms a salt bridge with Arg314 of a symmetry mate, but overall Glu332 in the T332E variant is more involved in crystal contact formation than Glu333 in the V333E variant (Supplementary Fig. S2).

### 3.5. Alternative conformations of entire loops in the B repeat

**Figure 7:**
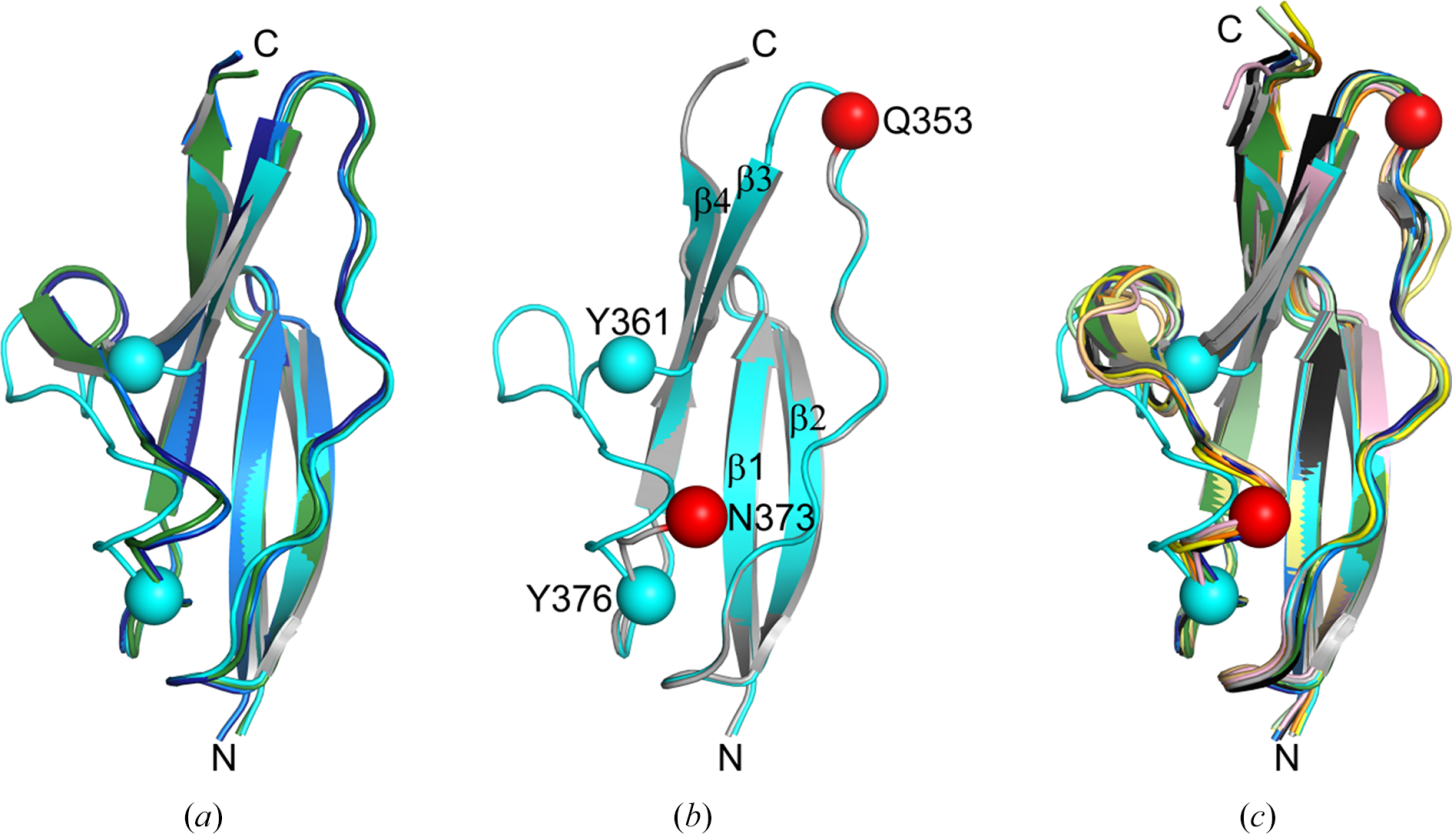
Flexibility of the β3–β4 loop in the B repeat. (*a*) Overlay of B repeats from the V333E variant (green) and T336Y chains *A* (dark blue), *B* (blue) and *C* (cyan). For T336Y chain *C*, C^α^ atoms of the first residue (Tyr361) and last residue (Tyr376) of the deviating loop are shown as cyan spheres. (*b*) Overlay of T336Y chain *C* (cyan) and wild-type InlB_392_ (gray; PDB entry 7pv9). For the wild type, C^α^ atoms of the last residue before and the first residue after the disordered region are shown as red spheres. (*c*) Overlay of all 13 B-repeat instances available in the PDB, each in a different color: PDB entries 2y5p (B repeat) chains *A*–*D*, 7nms (InlB_392_ T332E variant, crystal form II), 7pv8 (InlB_392_ T332E variant, crystal form I), 7pv9 (wild-type InlB_392_) chains *A*–*C*, 9qr4 (InlB_392_ T336Y variant) chains *A*–*C* and 9qr5 (InlB_392_ V333E variant).

In T336Y chain *C*, residues 361–376 adopt a conformation distinct from the other three B repeats described here (Fig. 7 ▸*a*). Electron density in this region is weak and suggests at least two conformations. We could model only one conformation, leaving residual difference density. In the related wild-type chain *C* (similar packing and domain orientation), there was no interpretable electron density for an even longer segment (residues 354–372) corresponding to strand β3 and the following loop in other B-repeat structures (Fig. 7 ▸*b*). An overlay of all B-repeat structures in the PDB indicates that the β3–β4 loop is the most flexible region (Fig. 7 ▸*c*). In both wild-type InlB_392_ and the T336Y variant, the chain *C* B repeats have the highest *B* factors (Supplementary Fig. S3). In both structures, the chain *C* B repeat contacts two further B repeats from symmetry-related chain *C* molecules, and this packing environment may contribute to the conformational heterogeneity in the β3–β4 loop.

## 4. Discussion

The V333E and T336Y variants were generated for functional assays rather than being designed to enhance crystallization (Bleymüller *et al.*, 2016 ▸), yet both yielded high-quality crystals. The improved diffraction properties are not merely an anecdotal finding because they are not due to crystal-to-crystal variation. For the wild type we had tested five crystals, of which only one diffracted to 3.3 Å resolution, while the other four diffracted poorly or not at all (Geerds *et al.*, 2022 ▸). For the V333E and T336Y variants, five and 15 data sets from different crystals had mean diffraction limits of 1.8 and 2.3 Å, respectively.

More intuitive and rational approaches to increase the crystallization propensity of a protein typically aim at obtaining a covalently and conformationally more homogenous sample. The mutations described here do not function by reducing conformational heterogeneity. The mutations are not located close to the linker connecting the internalin domain to the B repeat and they do not reduce the interdomain flexibility. Moreover, both mutations increase the surface entropy of the respective residue. The T336Y mutation might be expected to benefit crystallization, as tyrosine is enriched in protein–protein interfaces and crystal contacts relative to its occurrence on the protein surface (Lo Conte *et al.*, 1999 ▸; Ofran & Rost, 2003 ▸; Prasad Bahadur *et al.*, 2004 ▸; Bordner & Abagyan, 2005 ▸). Accordingly, newer variants of SER suggest replacing Lys or Glu not only by Ala, but also by Thr or Tyr (Cooper *et al.*, 2007 ▸; Derewenda, 2010 ▸). Tyr336 in the T336Y variant actually forms a (sometimes water-mediated) hydrogen bond to a neighboring molecule, which could not be formed by the native residue Thr336.

The positive effect of the T332E and V333E mutations was unexpected. Glutamate is generally considered to be dis­favored in protein–protein binding sites and crystal contacts and it has been reported to be the second most disfavored residue in protein–protein interfaces after lysine (Lo Conte *et al.*, 1999 ▸; Ofran & Rost, 2003 ▸; Prasad Bahadur *et al.*, 2004 ▸; Bordner & Abagyan, 2005 ▸). Lysine and glutamate are the only two amino acids whose frequency in the protein sequence was found to correlate negatively with successful crystal structure determination in a large-scale analysis (Price *et al.*, 2009 ▸). The T332E and V333E mutations would hence be expected to impede crystallization. Instead, the T332E variant showed the highest success rate in crystallization (Table 5 ▸) and the V333E variant resulted in the highest resolution structure. Several recent publications are in line with our finding that even mutations that increase the entropy of a surface residue can help crystallization (Naumov *et al.*, 2019 ▸; Schaefer *et al.*, 2024 ▸). These occasional observations of improved crystallization upon substituting a lower entropy residue by glutamate are theoretically supported by a very recent study that, contradicting earlier results, found glutamate and even lysine to be statistically overrepresented in crystal-packing interfaces (Banayan *et al.*, 2024 ▸).

It is difficult to rationalize the beneficial effect of the T332E and V333E mutations on crystallization. SER, which removes rather than introduces glutamates, is theoretically based on thermodynamic considerations (Longenecker *et al.*, 2001 ▸; Derewenda, 2004*b* ▸, 2007 ▸; Czepas *et al.*, 2004 ▸; Cieślik & Derewenda, 2009 ▸). Hence, removing surface glutamates is the more intuitive and rational choice than mutating small surface residues to glutamate. The results presented here could be an outlier, particularly given the limited scope of our report that covers one protein crystallized under a restricted set of conditions, which all use PEGs as precipitant. The unexpected success of the T332E and V333E mutations suggests that one should stay open-minded and that any crystallization strategy should be applied with caution, rather than being viewed as exclusive.

Our new structures of InlB_392_ reported here all form the previously observed crystal contact between B-repeat strand β2 and the interrepeat region of a neighboring molecule, confirming that this contact is energetically favorable. In all three variants with improved crystallization behavior for which we have determined the structure (T332E, V333E and T336Y), the mutation is located in this recurring crystal contact. Our previous report on the crystallization of the T332E mutant provided only an anecdotal hint that a point mutation within this crystal contact can improve the diffraction properties (Geerds *et al.*, 2022 ▸). This work, although not systematic, provides further examples that support this hypothesis by including the mutation of two additional residues. The new variants (T336Y, 1.60 Å resolution; V333E, 1.45 Å resolution) diffract even better than that the previously reported T332E variant (1.80 Å resolution). The recurring crystal contact forms regardless of whether a substitution has no effect on biological activity (V333E and T336Y) or whether it is a loss-of-function mutation (T332E) (Bleymüller *et al.*, 2016 ▸), suggesting that it does not represent a physiological protein–protein interaction. Neither the high interdomain flexibility of InlB_392_ nor high side-chain entropy appear to be the main reason for the poor crystallization propensity of wild-type InlB_392_. Variant D, which showed the second highest success rate of crystallization after the T332E variant (Table 5 ▸), contains mutations in strand β2 and one mutation (I334K) that increases the surface entropy. All variants with increased crystallization propensity or improved diffraction properties contain amino-acid exchanges in strand β2, while the multiple substitutions of variant A distant from strand β2 had no positive effect. Hence, particular surface properties of strand β2 within the B repeat might impede crystallization. This would be in line with the hypothesis that protein crystallization is hindered by negative evolutionary design and the resulting expectation that random mutations of surface amino acids that do not alter the protein structure would likely lead to a more crystallizable protein (Doye *et al.*, 2004 ▸).

## 5. Conclusion

Our work confirms that surface mutagenesis is a valuable tool to crystallize a protein of interest. It suggests that it might even be worthwhile to consider substitutions that increase the side-chain entropy of the mutated surface residue.

## Supplementary Material

PDB reference: *Listeria monocytogenes*internalin B, residues 36–392, T336Y variant, 9qr4

PDB reference: V333E variant, 9qr5

Diffraction images for T336Y variant.: https://doi.org/10.15785/SBGRID/1174

Diffraction images for V333E variant.: https://doi.org/10.15785/SBGRID/1175

Supplementary Figures. DOI: 10.1107/S2053230X25010416/va5067sup1.pdf

## Figures and Tables

**Table 1:** Macromolecule-production information The sequence of the GST-tag is not shown. Residues in italics do not derive from InlB, but from the expression vector. The recognition sequence of the 3C protease is underlined and its cleavage site is indicated by a vertical line. A single sequence is shown for the V333E and T336Y variants and the deviating residues are highlighted in bold in square brackets. The V333E variant contains the sequence ^333^EIKT^336^ and the T336Y variant contains ^333^VIKY^336^.

Source organism	*Listeria monocytogenes* EGDe
Expression vector	pGEX-6P-1 (Bleymüller *et al.*, 2016 ▸)
Expression host	*Escherichia coli* BL21
Complete amino-acid sequence of the construct produced	[GST-tag]-*SD**LEVLFQ*|*GP**LGS*ETITVPTPIKQIFSDDAFAETIKDNLKKKSVTDAVTQNELNSIDQIIANNSDIKSVQGIQYLPNVTKLFLNGNKLTDIKPLANLKNLGWLFLDENKVKDLSSLKDLKKLKSLSLEHNGISDINGLVHLPQLESLYLGNNKITDITVLSRLTKLDTLSLEDNQISDIVPLAGLTKLQNLYLSKNHISDLRALAGLKNLDVLELFSQECLNKPINHQSNLVVPNTVKNTDGSLVTPEIISDDGDYEKPNVKWHLPEFTNEVSFIFYQPVTIGKAKARFHGRVTQPLKEVYTVSYDVDGT**[V/E]**IK**[Y/T]**KVEAGTRITAPKPPTKQGYVFKGWYTEKNGGHEWNFNTDYMSGNDFTLYAVFKAET

**Table 2:** Crystallization

Protein	InlB_392_, T336Y variant	InlB_392_, V333E variant
Method	Sitting-drop vapor diffusion	Sitting-drop vapor diffusion
Plate type	96-well, SWISSCI MRC 2 lens	96-well, SWISSCI MRC 2 lens
Temperature (K)	277	277
Protein concentration (mg ml^−1^)	10	20
Buffer composition of protein solution	10 m*M* Tris pH 8.0, 20 m*M* NaCl	10 m*M* Tris pH 8.0, 20 m*M* NaCl
Composition of reservoir solution	MORPHEUS screen, condition F3: 0.1 *M* mixture of imidazole and MES (acid) pH 6.5, 10% PEG 4000, 20% glycerol, 0.02 *M* of each monosaccharide (D-glucose, D-mannose, D-galactose, L-fucose, D-xylose and *N*-acetyl-D-glucosamine)	PEG smear screen MMW, condition E1: 0.1 *M* HEPES pH 7.5, 22.5% PEG medium-molecular-weight (MMW) mixture consisting of PEGs 1500, 2000, 2000 MME, 3000, 3350, 4000 and 5000 MME
Volume and ratio of drop	1 µl + 1 µl	200 nl + 100 nl
Volume of reservoir (µl)	70	70

**Table 3:** Data collection and processing Values in parentheses are for the outer shell.

	InlB_392_, T336Y variant	InlB_392_, V333E variant
DOI of diffraction images	http://doi.org/10.15785/SBGRID/1174	http://doi.org/10.15785/SBGRID/1175
Diffraction source	BL14.2, BESSY II	P13, PETRA III
Wavelength (Å)	0.9184	0.9763
Temperature (K)	100	100
Detector	PILATUS 2M	PILATUS 6M-F
Crystal-to-detector distance (mm)	176.4	269
Rotation range per image (°)	0.1	0.1
Total rotation range (°)	360	360
Exposure time per image (s)	0.4	0.0377
Space group	*P*2_1_2_1_2_1_	*P*2_1_2_1_2_1_
*a*, *b*, *c* (Å)	44.92, 149.17, 220.81	41.41, 90.77, 99.12
α, β, γ (°)	90.00, 90.00, 90.00	90.00, 90.00, 90.00
Mosaicity (°)	0.168	0.077
Resolution range (Å)	50–1.60 (1.64–1.60)	50–1.45 (1.49–1.45)
Total No. of reflections	2563920 (172156)	870129 (58484)
No. of unique reflections	192399 (13816)	67167 (4910)
Completeness (%)	98.0 (96.4)	100.0 (99.9)
Multiplicity	13.33 (12.46)	12.95 (11.91)
〈*I*/σ(*I*)〉	19.04 (2.00)	17.81 (1.95)
*R*_meas_ (%)	7.2 (159.2)	7.7 (133.8)
CC_1/2_	0.999 (0.627)	0.999 (0.757)
Overall *B* factor from Wilson plot (Å^2^)	33.0	25.9

**Table 4:** Structure solution and refinement Values in parentheses are for the outer shell.

	InlB_392_, T336Y variant	InlB_392_, V333E variant
PDB code	9qr4	9qr5
Resolution range (Å)	20.13–1.60 (1.62–1.60)	45.38–1.45 (1.47–1.45)
Completeness (%)	97.97 (94.39)	99.96 (99.79)
No. of reflections		
Refinement	192169 (6057)	67074 (2873)
Working set	182520 (5731)	63785 (2728)
Test set	9649 (326)	3289 (145)
Final *R*_cryst_	0.1728 (0.2854)	0.1550 (0.2884)
Final *R*_free_	0.1959 (0.3018)	0.1787 (0.3562)
No. of non-H atoms		
Protein	8674	2886
Ligand	66	18
Water	1420	575
Total	10160	3479
R.m.s. deviations		
Bond lengths (Å)	0.008	0.012
Angles (°)	0.87	1.19
Average *B* factors (Å^2^)		
Overall	37.79	23.65
Protein	36.86	21.38
Ligand	56.84	44.26
Water	42.61	34.44
Ramachandran plot		
Most favored (%)	97.09	97.74
Allowed (%)	2.81	2.26
Outlier (%)	0.09	0.00
Clashscore	0.79	1.02

**Table 5:** Outcome of crystallization screens with six variants of InlB_392_ Variants A, C and D (Bleymüller *et al.*, 2016 ▸) contain multiple substitutions as mentioned in the text. Crystallization hits include microcrystals and needles. n.d., not determined (these plates were not set up, for example due to limitations in protein supply or failure of the crystallization robot).

		No. of conditions with crystals								
		MORPHEUS		PEG smear HMW/MMW		PEG smear LMW/BMW				
	Protein concentration (mg ml^−1^)	4°C	20°C	4°C	20°C	4°C	20°C	Total No. of conditions	Success rate (%)	Best resolution (Å)
Wild type	10	1	1	0	n.d.	n.d.	n.d.	288	0.7	3.30
	20	n.d.	n.d.	n.d.	n.d.	0	0	192	0.0	n.d.
T332E	10	10	20	7	21	16	22	576	16.7	1.85
V333E	20	n.d.	n.d.	1	0	0	0	384	0.3	1.45
T336Y	10	3	1	1	1	n.d.	1	480	1.3	1.60
Variant A	22	0	0	0	0	0	0	576	0.0	n.d.
Variant C	20	n.d.	n.d.	0	0	0	0	384	0.0	n.d.
Variant D	10	3	13	7	36	4	20	576	14.4	n.d.
	20	n.d.	n.d.	4	13	2	8	384	7.0	3.30
