# Supplementary material for: Single mutations to tyrosine or glutamate improve the crystallizability and crystal diffraction properties of a flexible two-domain protein
Source: Acta Crystallogr F Struct Biol Commun. 2026 Jan 1;82(Pt 1):4–13. doi: 10.1107/S2053230X25010416 (PMC12809440; doi:10.1107/S2053230X25010416)
Supplement: Supplementary file 1 [file f-82-00004-sup1.pdf]

Acta Cryst F

STRUCTURAL BIOLOGY COMMUNICATIONS

Volume 81 (2025)

Supporting information for article:

Single mutations to tyrosine or glutamate improve the crystallizability and crystal diffraction properties of a flexible two-domain protein

Christina Geerds and Hartmut H. Niemann

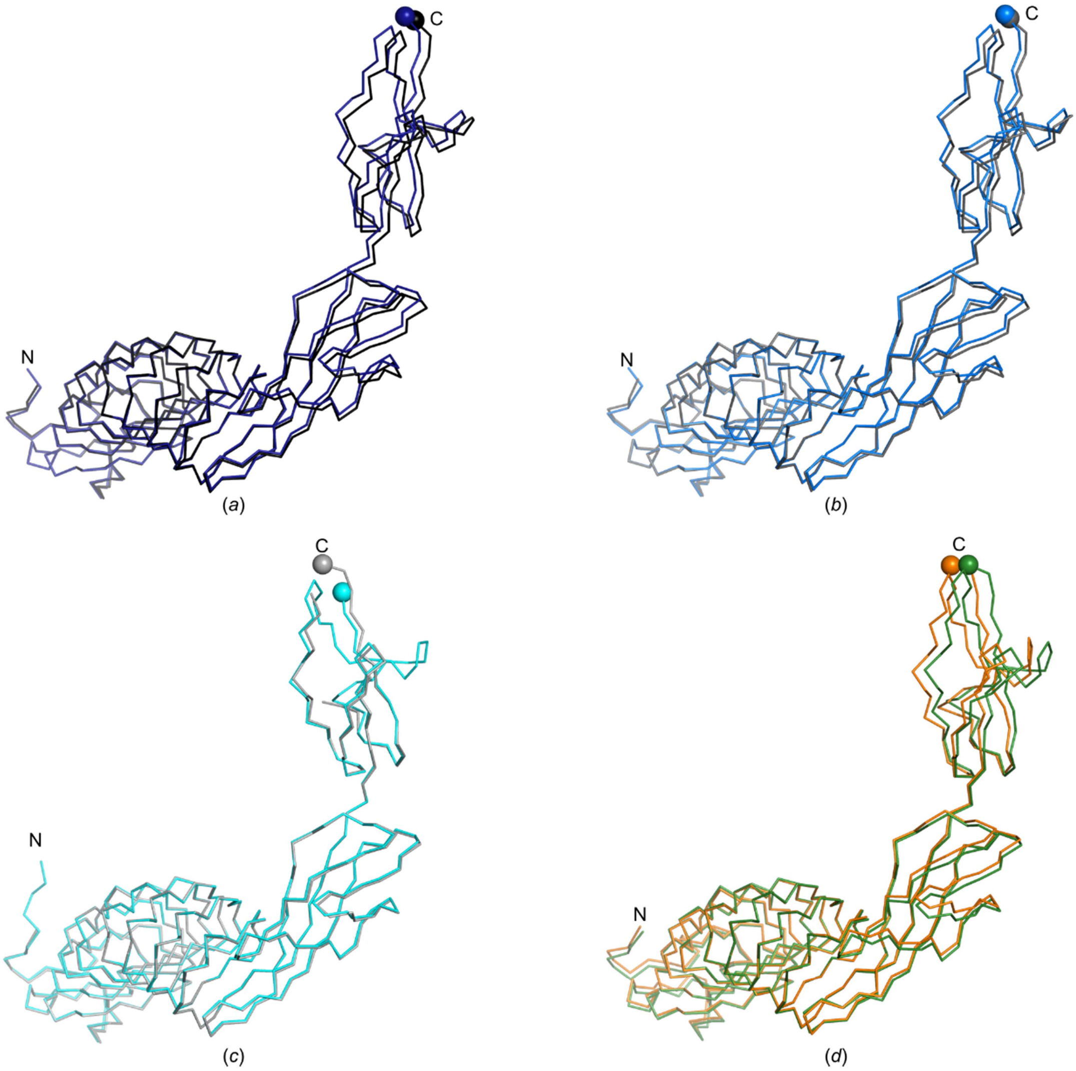

Figure S1 Overlay of $InlB_{392}$ structures reported here with related chains from previously published structures with similar crystal packing. Superposition was performed on the leucine rich repeat region of the internalin domain (residues 80-240) to highlight relative movements of the B repeat. The C-terminal residue of each structure is indicated by a C^{α} sphere. (*a*) $InlB_{392}$_T336Y chain *A* (dark blue) aligned with wild type $InlB_{392}$ chain *A* ($InlB_{392}$_wt; PDB entry 7pv9). (*b*) $InlB_{392}$_T336Y chain *B* (blue) aligned with $InlB_{392}$_wt chain *B*. (*c*) $InlB_{392}$_T336Y chain *C* (cyan) aligned with $InlB_{392}$_wt chain *C*. (*d*) $InlB_{392}$_V333E (green) aligned with $InlB_{392}$_T332E crystal form II (PDB entry 7nms).

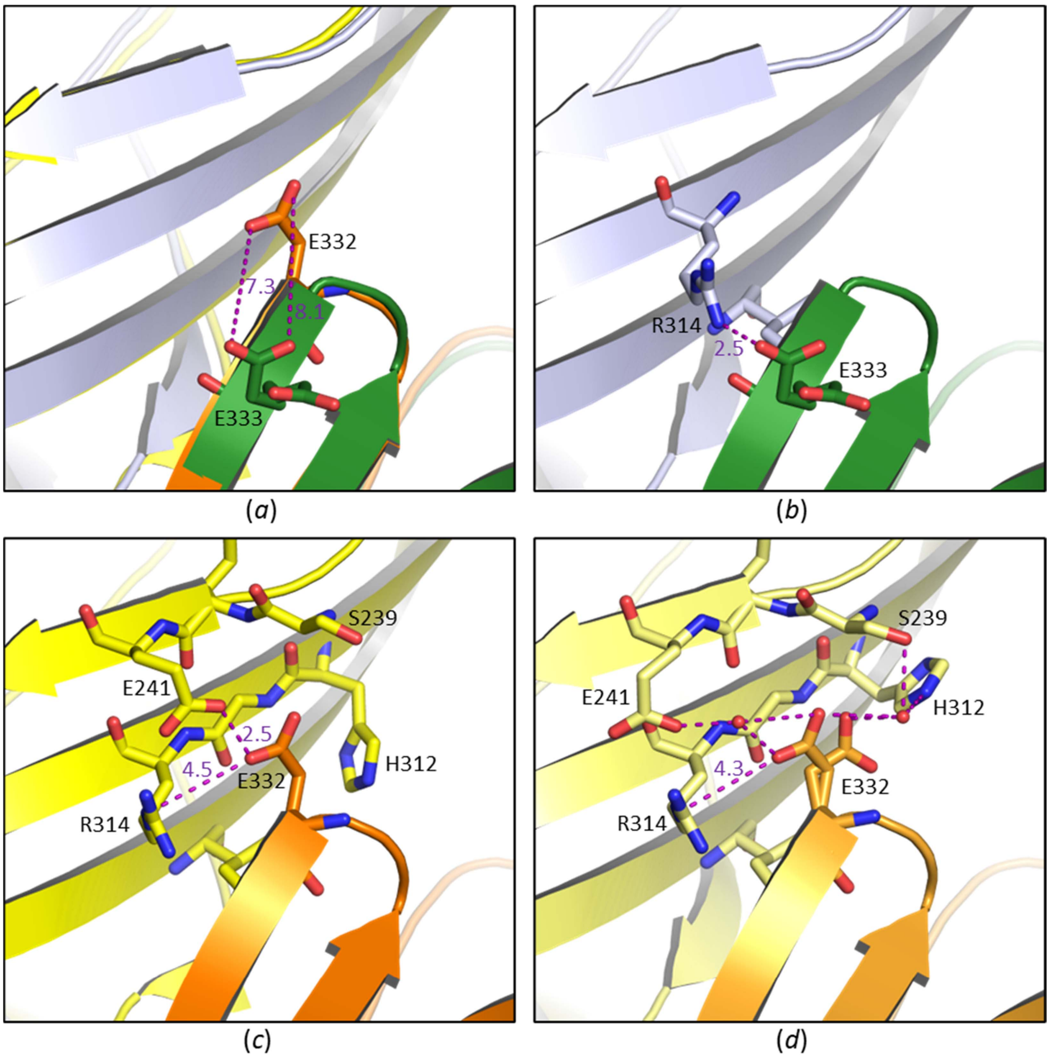

Figure S2 Participation of the introduced E332 or E333 in the conserved crystal contact. (*a*) Overlay of T332 crystal form II (PDB entry 7nms; orange) and V333E (green) on their B repeats. E332 and E333 are located on different sides of strand β2. E332 is in close contact with the IR region of its symmetry mate (yellow), while E333 is distant from the IR region of its symmetry mate (light blue). (*b*) All residues of the symmetry mate within 4 Å of E333 in V333E are shown as sticks with light blue carbon atoms. The salt bridge to R314 is indicated as dashed purple line and its distance is given in Å. (*c*) All residues of the symmetry mate within 4 Å of E332 (orange) in T332E crystal form II (PDB entry 7nms) are shown as sticks with yellow carbon atoms. Polar contacts are indicated as dashed purple lines. The distance of these contacts is given in Å. (*d*) Crystal contact in non-isomorphous crystal form I of T332E. All residues of the symmetry mate within 4 Å of E332 (light orange) in T332E crystal form I (PDB entry 7pv8) are shown as sticks with pale yellow carbon atoms. Bridging waters are shown as red spheres. Polar contacts are indicated as dashed purple lines. For clarity, only the distance of the salt bridge to R314 is given in Å.

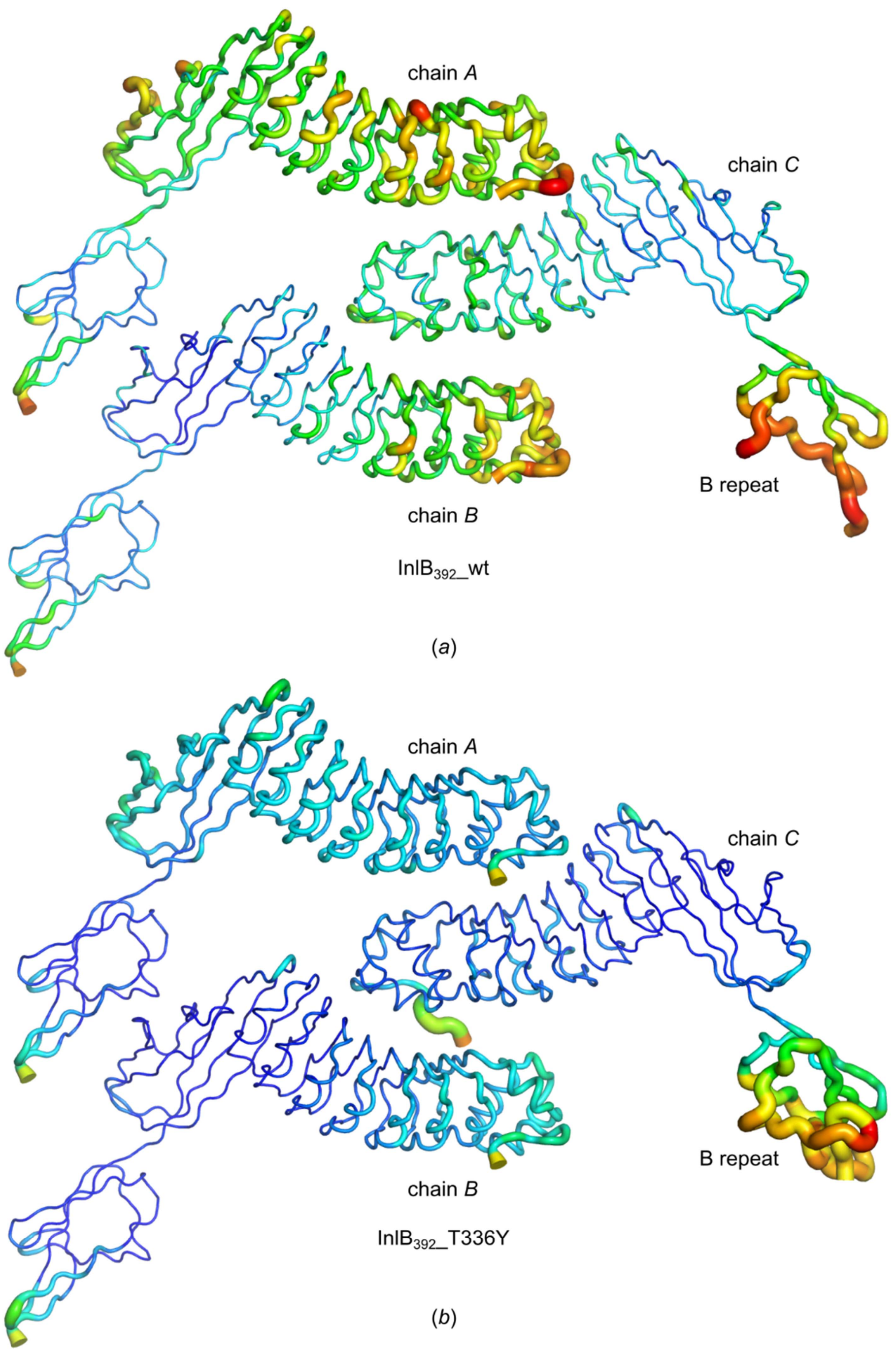

Figure S3 B-factor distribution in $InlB_{392}$ wild type and T336Y. (*a*) $InlB_{392}$_wt (PDB entry 7pv9). (*b*) $InlB_{392}$_T336Y (PDB entry 9qr4).
